# 2-(3-(4-Bromophenyl)-5-{3-[5-methyl-1-(4-methylphenyl)-1*H*-1,2,3-triazol-4-yl]-1-phenyl-1*H*-pyrazol-4-yl}-4,5-dihydro-1*H*-pyrazol-1-yl)-4-(4-chlorophenyl)-1,3-thiazole. Corrigendum

**DOI:** 10.1107/S2414314626000994

**Published:** 2026-02-13

**Authors:** Gamal A. El-Hiti, Bakr F. Abdel-Wahab, Rizk E. Khidre, Mohamed S. Mostafa, Amany S. Hegazy, Benson M. Kariuki

**Affiliations:** a Cornea Research Chair, Department of Optometry, College of Applied Medical Sciences, King Saud University, PO Box 10219, Riyadh 11433, Saudi Arabia; b Department of Chemistry, College of Science and Humanities, Shaqra University, Duwadimi, Saudi Arabia; c Applied Organic Chemistry Department, National Research Centre, Dokki 12622, Giza, Egypt; d Chemistry Department, Faculty of Science, Jazan University, Jazan 2079, Saudi Arabia; e Chemical Industries Division, National Research Centre, Dokki 12622, Giza, Egypt; f Chemistry Department, Faculty of Science, Damietta University, Egypt; g School of Chemistry, Cardiff University, Main Building, Park Place, Cardiff CF10 3AT, UK

**Keywords:** crystal structure, thia­zole, pyrazole, 2.2.3-triazole

## Abstract

In the paper by El-Hiti *et al.* [*IUCrData* (2018), **8**, x180443], there was an error in the title with respect to the chemical name.

## Abstract 2

Corrigendum to *IUCrData* (2018), **3**, x180443.

## Graphical abstract

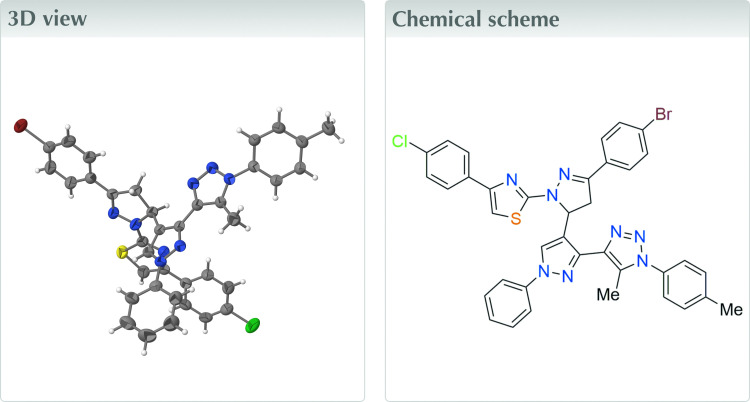

<!?tpt=-20pt>The chemical name of the title compound in the paper by El-Hiti *et al.* (2018 ▸) is incorrect and should be ‘2-(3-(4-Bromophenyl)-5-{3-[5-methyl-1-(4-methylphenyl)-1*H*-1,2,3-triazol-4-yl]-1-phenyl-1*H*-pyrazol-4-yl}-4,5-dihydro-1*H*-pyrazol-1-yl)-4-(4-chlorophenyl)-1,3-thiazole’, as given above. The chemical scheme is also corrected

## Supplementary Material

Supporting information file. DOI: 10.1107/S2414314626000994/me6367Isup1.cml
